# Idéfix: identifying accidental sample mix-ups in biobanks using polygenic scores

**DOI:** 10.1093/bioinformatics/btab783

**Published:** 2021-11-18

**Authors:** Robert Warmerdam, Pauline Lanting, Lifelines Cohort Study, Patrick Deelen, Lude Franke

**Affiliations:** Department of Genetics, University Medical Center Groningen, University of Groningen, 9700AB Groningen, The Netherlands; Department of Genetics, University Medical Center Groningen, University of Groningen, 9700AB Groningen, The Netherlands; Department of Genetics, University Medical Center Groningen, University of Groningen, 9700AB Groningen, The Netherlands; Department of Genetics, University Medical Center Utrecht, 3508GA Utrecht, The Netherlands; Department of Genetics, University Medical Center Groningen, University of Groningen, 9700AB Groningen, The Netherlands

## Abstract

**Motivation:**

Identifying sample mix-ups in biobanks is essential to allow the repurposing of genetic data for clinical pharmacogenetics. Pharmacogenetic advice based on the genetic information of another individual is potentially harmful. Existing methods for identifying mix-ups are limited to datasets in which additional omics data (e.g. gene expression) is available. Cohorts lacking such data can only use sex, which can reveal only half of the mix-ups. Here, we describe *Idéfix*, a method for the identification of accidental sample mix-ups in biobanks using polygenic scores.

**Results:**

In the Lifelines population-based biobank, we calculated polygenic scores (PGSs) for 25 traits for 32 786 participants. We then applied *Idéfix* to compare the actual phenotypes to PGSs, and to use the relative discordance that is expected for mix-ups, compared to correct samples. In a simulation, using induced mix-ups, *Idéfix* reaches an AUC of 0.90 using 25 polygenic scores and sex. This is a substantial improvement over using only sex, which has an AUC of 0.75. Subsequent simulations present *Idéfix’s* potential in varying datasets with more powerful PGSs. This suggests its performance will likely improve when more highly powered GWASs for commonly measured traits will become available. *Idéfix* can be used to identify a set of high-quality participants for whom it is very unlikely that they reflect sample mix-ups, and for these participants we can use genetic data for clinical purposes, such as pharmacogenetic profiles. For instance, in Lifelines, we can select 34.4% of participants, reducing the sample mix-up rate from 0.15% to 0.01%.

**Availabilityand implementation:**

*Idéfix* is freely available at https://github.com/molgenis/systemsgenetics/wiki/Idefix. The individual-level data that support the findings were obtained from the Lifelines biobank under project application number ov16_0365. Data is made available upon reasonable request submitted to the LifeLines Research office (research@lifelines.nl, https://www.lifelines.nl/researcher/how-to-apply/apply-here).

**Supplementary information:**

Supplementary data are available at *Bioinformatics* online.

## 1 Introduction

Biobanks systematically collect (human) biological samples and associated data for research purposes. Enrichment of these biobanks is common through measurements on the collected samples, for example by analyzing serum levels of biological compounds or by genotyping. Repurposing of genetic data for clinical use has become of increasing interest over the past decades (National Academies of Sciences, Engineering, and Medicine *et al.*, 2018). Whereas some initiatives make use of existing diagnostic data (Lee *et al.*, 2020), there are great opportunities for clinical repurposing of existing non-diagnostic data generated by biobanks. However, quality control measures to prevent sample mix-ups (or sample swaps) are commonly less stringent because of the research setting this data was generated in. The existence of sample mix-ups can result in the reporting of results that don’t correspond with the individual and could thereby harm this individual’s health (Ciszkowski *et al.*, 2009; Gasche *et al.*, 2004; Heemskerk-Gerritsen *et al.*, 2019). The number of sample swaps that are expected in a research setting varies, with an average having been reported of 3% in genomics datasets, whereas the frequency of misidentification in laboratory diagnostics is estimated to be between 0.01% and 0.1% (Lippi *et al.*, 2017).

Identifying sample mix-ups in biobank data is a prerequisite to allow it to be used clinically and helps to improve the quality of the data for scientific analyses that will benefit from improved statistical power. For example, it is known from simulations that sample mix-ups negatively affect the power of genome-wide association studies (GWASs) and thereby hinder the detection of variants with smaller effect sizes (Buyske *et al.*, 2009; Ho and Lange, 2010; Samuels *et al.*, 2009; Zheng and Tian, 2005).

The most basic test for sample mix-up identification consists of a comparison of the reported sex of individuals and the inferred sex based on the genetic data of these individuals. A drawback of this sex correspondence check is that it is unable to detect sample mix-ups between samples of the same sex. Furthermore, the sex concordance check is only applicable to datasets containing both sexes and is only able to identify up to 50% of mix-ups given that the dataset contains an equal number of males and females. Pedigree information can also be utilized if family members are included in the biobank and the family relationships are known. In addition, self-reported ethnicities can potentially aid in the identification of mix-ups, although self-reported or observer-reported ethnicities are not always reliable, especially in the case of mixed ancestry (Dumitrescu *et al.*, 2010; Tzvetkov *et al.*, 2010). A variety of more complex methods have been developed in the past that are able to detect and potentially correct sample mix-ups reliably with the presence of omics datasets (Du *et al.*, 2017; Jiang *et al.*, 2020; Westra *et al.*, 2011). As with the sex correspondence check, these methods rely on determining whether a phenotype, for example, the expression of genes, corresponds to the expected phenotype based on the individual’s genotype. An observed mismatch adds evidence for the sample being a mix-up. Drawbacks of the latter methods are that they either require specific data (such as gene expression or methylation data) that are not commonly available.

In recent years, GWASs have been increasingly powerful, aiding the power and reliability of polygenic scores (PGSs) (Dudbridge, 2013). These PGSs can be used to predict the phenotypes of individuals even if they were not part of the original GWAS. Since PGSs represent an individual’s propensity to a phenotype based on their genetic makeup, they may be useful for identifying sample mix-ups as these PGSs provide an additional method for determining whether the sample’s phenotype corresponds to the sample’s genotype. Here, we describe a PGS-based sample mix-up identification method (*Idéfix)* that combines PGSs from multiple traits and determines per sample whether the predicted phenotypes conform to the observed phenotypes, enabling the identification of sample mix-ups. We show our method has predictive power for differentiating between correct samples and sample mix-ups, and that through performing such quantification for a sufficient number of phenotypes we can reliably identify and remove sample mix-ups.

## 2 Materials and methods

### 2.1 Datasets

We used adult samples from the Lifelines prospective follow-up biobank (Stolk *et al.*, 2008). These samples were genotyped using the Infinium Global Screening Array_**^®^**_ (GSA) MultiEthnic Disease Version 1.0. Within these samples, we developed and implemented a method to identify sample mix-ups. To do so, we selected 25 traits for which large-scale GWASs have previously been performed, for which the accompanying summary statistics are available, and for which the specific phenotypes were measured in Lifelines (Demenais *et al*., 2018, Evangelou *et al*., 2018, Hoffmann *et al*., 2018, Lee *et al*., 2018, Mahajan *et al*., 2018, Van der Harst and Verweij, 2018, Vuckovic *et al*., 2020, Wheeler *et al*., 2017, Wuttke *et al*., 2019, Yengo *et al*., 2018). The selected traits and corresponding GWASs are listed in Supplementary Table S1.

### 2.2 Calculating PGSs

For developing and executing the mix-up identification method, PGSs had to be calculated first. A variety of methods and algorithms have been developed over the past years. A recent addition to this range of methods on polygenic prediction is PRS-CS, a tool with superior reported accuracy that functions by inferring posterior effect sizes of single nucleotide polymorphisms (SNPs) (Ge *et al.*, 2019). The method has been shown to equal or outperform competing methods with various GWASs (Chun *et al.*, 2020). Therefore, this tool was chosen to calculate PGSs. GWAS summary statics were processed to comply with the PRS-CS input format. For this, we added reference SNP identifiers (RSIDs) to the summary statistics when these were not initially present. This was done by matching the genomic locations of variants to variants from dbSNP (build 137).

The genetic data was preprocessed using PLINK 2.0 (Chang *et al.*, 2015; Purcell and Chang), by excluding ambiguous SNPs and converting the genotype data from VCF to the hybrid PLINK 2.0 bpgen format, maintaining allelic dosage information. Variants were removed if either their minor allele frequency (MAF) was less than 0.01, imputation score was below 0.3 or missing call rates exceeded 0.25. Subsequently, PRS-CS was run to calculate effect sizes given the GWAS summary statistics. We used the European reference linkage disequilibrium (LD) panel provided by the PRS-CS authors. Other parameter settings were left as default. PLINK 2.0 was used to sum variant dosages weighted by the posterior effect sizes as calculated by PRS-CS. The performance of PGSs for continuous traits were assessed by calculating the proportion of the variance in actual phenotypes that is explained by these PGSs ($R^{2}$**)**. For ordinal or binary phenotypes, we calculated the area under the ROC curve (AUC). For testing the performance of PGSs all phenotypes were corrected for age, sex and their interaction effects. $R^{2}$-values were compared with those reported by literature (Supplementary Table S2).

### 2.3 Processing of phenotypes

The phenotypes were processed to match the design of the corresponding GWAS, for example exclusion of samples with certain comorbidities or log transformation. Exclusion criteria and transformations applied are presented in Supplementary Table S3. Estimated glomerular filtration rate (eGFR) was calculated with the CKD-EPI (Levey *et al.*, 2009). Coronary artery disease was defined as having had self-reported balloon angioplasty, bypass surgery or a heart attack. Self-reported level of education of Lifelines participants was converted to the number of years of US schooling according to the mapping in Supplementary Table S4 (Okbay *et al.*, 2016). The characteristics of the processed study population is presented in Supplementary Table S5.

### 2.4 Identifying sample mix-ups

Sample mix-up procedures rely on the (lack of) concordance of an individual’s measured phenotype with their predicted phenotype based on the individual’s genotype. Our method extends this concept by using PGSs to predict an individual’s phenotype. The calculated PGSs are expected to be reasonably accurate for traits like height, where a larger than expected deviance of the predicted phenotype from the measured phenotype adds evidence for a sample being mixed-up. Since the discordance between predicted and measured phenotypes is expected to be relatively high for mix-ups, and relatively low for correct samples, adding additional traits will add predictive power to identify sample mix-ups.

**Fig. 1.**
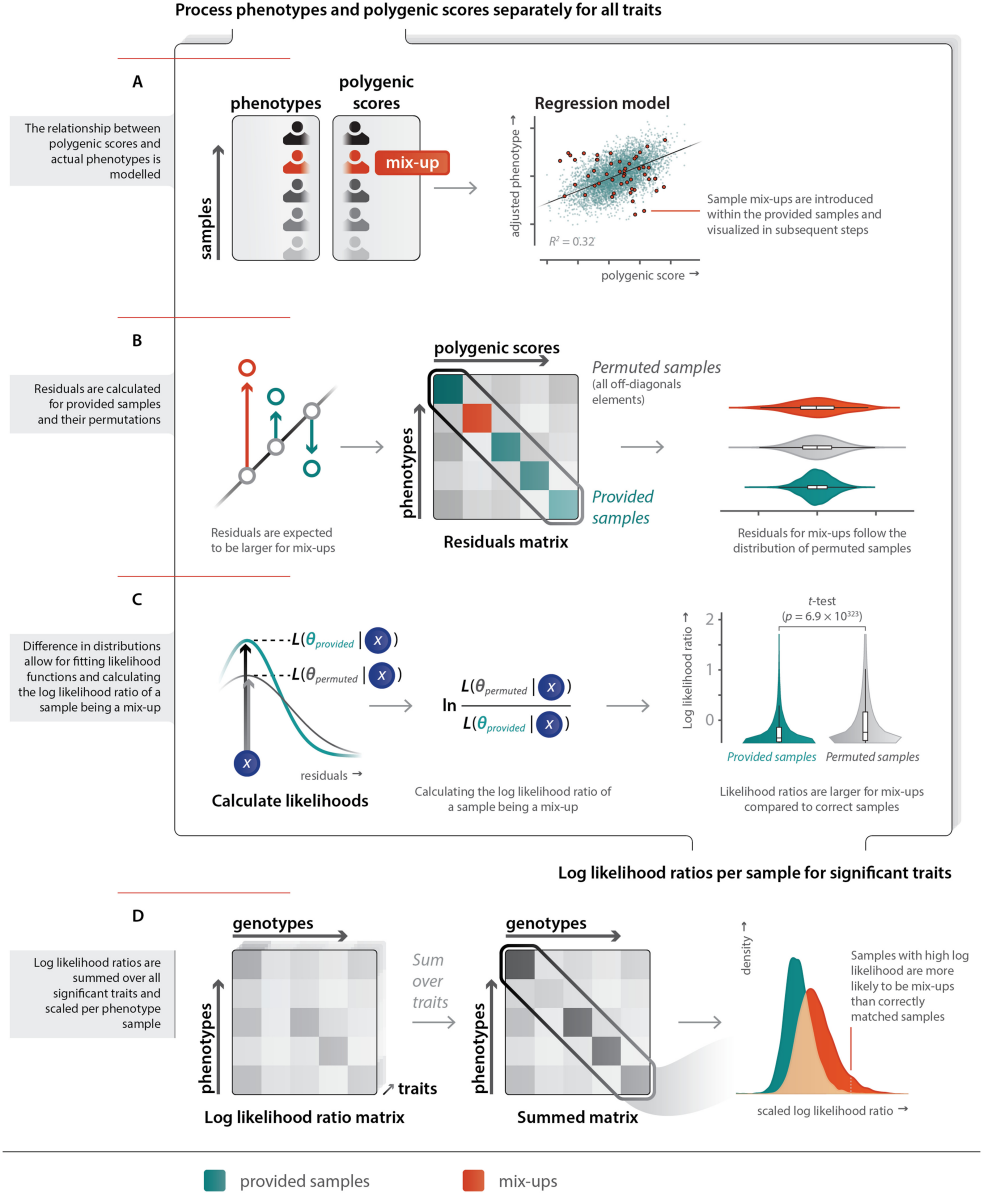
Method overview that indicates how PGSs and measured phenotypes are used to identify mix-ups. The steps A, B and C are performed separately for all traits. The scatterplot in A, and the distributions visualized in B, C and D are generated using a subset of 5120 samples from the Lifelines dataset of which 1% was introduced as a fake sample mix-up (shown in red). (**A**) The relationship between the input variables is modeled together in a linear model for a continuous trait. This is shown on the right. Introduced fake mix-ups are shown in red. (**B**) Residuals are calculated using the previously fitted model for both the provided sample mappings (main diagonal of the plotted matrix) and the permuted samples (off diagonal in the plotted matrix). The violin plots on the right indicate that permuted samples (grey) and mix-ups (red) are similarly distributed and differ from the residuals for the provided sample mappings (green). (**C**) (left) For continuous traits, Gaussian functions are fitted to the permuted (grey) and provided sample mappings (green) to calculate the likelihood of a residual fitting better in the correct or mix-up residual distributions. (middle) Dividing the likelihoods and log-transforming the results in log likelihood ratios of a sample being a mix-up (LLRs). (right) A *t*-test is used to test if there is a significant difference between LLRs for permuted and provided sample mappings. (**D**) The matrices on the left and middle indicate summing LLRs over significant traits, and that this aids the predictive power of LLRs. The densities on the right indicate the predictive power of LLRs scaled per row of the LLR matrix

An overview of our method is illustrated in Figure  1. The first steps are performed separately for every included trait. As indicated in Figure  1A, initially, the relationship between PGSs and actual phenotypes are modeled for the samples that are considered to be correct. For binary traits, like red hair color or the presence of a medical condition, this relationship is modeled using logistic regression. An ordered logistic regression is applied to ordinal traits, which for our set of phenotypes is limited to the spectrum of hair colors ranging from black through brown to blonde. For quantitative traits, a linear model is used. The summary statistics that are used for calculating PGSs are not able to explain all variation in the phenotype. This is partly due to non-genetic effects. Another cause is that in GWASs, variables that covary with phenotypes, such as age and sex, are often corrected for. The effects of these covariates therefore are not reflected in the summary statistics. To account for the remaining variation that the summary statistics are not able to explain, the predicted phenotypes in our method are modeled using age, sex and their interaction effect as covariates.

In the following step, for every sample the residuals are calculated, both for the samples that are assumed to be correct—given the provided sample mapping—and their permutations. Here, the residuals are essentially the phenotypic variance that cannot be explained by the PGS of a sample. It is therefore expected that the permuted samples have higher residuals compared with correct phenotype-genotype mappings. Permuted samples consist of all other combinations of genotypes and phenotypes, as indicated in Figure  1B. In this figure, the different sample mappings are illustrated in a matrix with the measured phenotype part of the samples across the rows, and genotype (PGS) part of the sample mappings across the columns. The main diagonal herein represents the provided sample mappings, with the off-diagonal representing permuted sample mappings. The figure also shows that the residuals of the provided sample mappings are differently distributed compared with those of both the provided sample mappings and the introduced fake mix-ups.

In case a linear model was used to model the relationship between the PGSs, covariates and the measured phenotypes, residuals are calculated as the deviations of the measured phenotypes from the fitted function. For a logistic regression model, deviance residuals are used to represent residuals which are calculated according to Equation (1). Herein, $d_{i}$ is defined as the deviance residual, $Y_{i}$ represents the observed outcome and $P_{i}$ represents the model’s predicted probability for the observed outcome. In case, an ordinal logistic regression model is used, an adaptation of this formula is used to deal with the increase in number of categories.

(1) di=-2ln⁡(Pi), Yi=1--2ln⁡(Pi), Yi=0.

We leverage the different distributions of residuals to calculate log likelihood ratios (LLRs) that indicate whether a sample is likely to be a mix-up or not. These LLRs are calculated on the ratio of the likelihood of a residual fitting in the distribution of provided sample mappings and the likelihood of a residual fitting in the distribution of permuted sample mappings. This step is illustrated in Figure  1C. Since residuals are expected to be normally distributed for continuous traits, likelihoods can be calculated using densities of Gaussian functions fitted to both of the residual distributions. This might however not hold for each trait. We, therefore, ascertained how the performance of this method compared to both an *equal width interval-discretization* and *equal frequency interval-discretization* technique with varying numbers of samples per bin.

*Equal width interval-discretization* was implemented to separate the parameter space corresponding to the residuals for the provided samples in bins so that each bin corresponds to an interval of equal width. The bins residing at both upper and lower limits of the parameter space are forced to include a minimum number of samples to prevent the model from overfitting and suffering from outliers having a large effect on separating residuals into bins. This minimum number of samples is also used to prune other bins that do not comply with this lower boundary. Pruned bins are split at their midpoint with the two halves joined to their respective nearest bin. *Equal frequency interval-discretization* was implemented to separate the parameter space corresponding to the residuals for the provided samples in bins so that each bin consisted of an equal number of values.

For both discretization techniques, the breaks obtained within the set of provided samples are subsequently applied to the residuals of permuted samples.

Discretization techniques and the Gaussian likelihood method were assessed by sampling 10 080 samples from the Lifelines dataset and artificially inducing 1% of mix-ups in these subsets. For each subset we applied each discretization technique using 20, 30, 50 and 80 average samples per bin. For the *equal width interval-discretization* the minimum number of samples per bin was set to 10. Corresponding LLRs can be represented as a matrix wherein rows represent phenotype parts of the sample mappings, and columns represent the genotype (PGS) part of the sample mappings. Within this matrix, LLRs were scaled over all rows, or phenotypes. We executed this procedure for 50 individual subsets. Analyses of variance (ANOVAs), followed by a number of Tukey’s honestly significant difference tests indicated that for continuous traits, the Gaussian likelihood method outperformed other methods in which a discretization approach was used (Supplementary Fig. S1).

Phenotypes of binary and ordinal traits are by definition not continuous, and residuals are not normally distributed as is the case for continuous traits. Therefore, using a Gaussian function for calculating likelihoods is inappropriate, and the *equal width interval-discretization* technique with an average of 80 samples per bin is used instead. Furthermore, we were not able to identify a significant advantage for the binary and ordinal traits for one of the likelihood models. Finally, this equal width interval-discretization was chosen over other discretization techniques because it should be less prone to overfitting due to the larger average sample size. Log likelihood ratios are calculated separately for each observed category within an ordinal trait.

We select the traits with significant predictive power for sample mix-ups using a *t*-test to determine whether LLRs of the provided sample mappings differ significantly from those of the permuted sample mappings per trait. In the final step, LLRs are summed over all traits that have a significant difference in the LLRs, resulting in a final matrix of LLRs. Summed LLRs are scaled per sample over the phenotypes. In the following section, we highlight a method for determining how an appropriate threshold can be obtained.

Using *Idéfix*, it is possible to select a subset of samples that adhere to a specified maximum mix-up rate. First, we estimate the expected number of mix-ups using the number of samples that fail a sex-check. Using the provided sample mappings and the permuted sample mappings the sensitivity for every provided sample mapping can be calculated. We then need to estimate the total number of expected mix-ups using the number of mix-ups that are identified using a sex-correspondence check, while accounting for the ratio of males and females in the cohort. This is shown in Equation (2).

(2) $${mr}_{\mathrm{estimated}}=\left(\frac{{\mathrm{swaps}}_{\mathrm{sex}¯\mathrm{check}}}{2({(1-f}_{m})\times f_{m})}-{\mathrm{swaps}}_{\mathrm{total}}\right)\frac{1}{N}.$$

Herein, ${mr}_{\mathrm{estimated}}$ denotes the estimated mix-up rate after excluding samples based on the sex-check, ${\mathrm{swaps}}_{\mathrm{sex}¯\mathrm{check}}$ denotes the number of sample mappings that fail a sex correspondence check, and $f_{m}$ denotes the observed frequency of males in the dataset. ${\mathrm{swaps}}_{\mathrm{total}}$ depicts the total number of samples that have been removed after the sex-check as well as other checks for sample mix-ups such as a pedigree check. N represents the total number of remaining samples after excluding the detected mix-ups.

Equation (3) shows how an updated estimated mix-up rate, denoted by ${mr}_{p\mathrm{ass}}$, is calculated for the set of samples that meet a certain sensitivity threshold.

(3) $${mr}_{\mathrm{pass}}={mr}_{\mathrm{estimated}}\times \mathrm{sensitivity}\frac{N}{n_{\mathrm{pass}}}.$$

Herein, ${mr}_{\mathrm{estimated}}$ denotes the original estimated mix-up rate calculated in Equation (2), $n_{\mathrm{pass}}$ denotes the number of samples that pass this threshold, and N represents the number of samples that were assessed.

For each sample, the ${mr}_{\mathrm{pass}}$ can be calculated. Samples for which the calculated ${mr}_{\mathrm{pass}}$ is below the required mix-up rate are marked to pass *Idéfix*. This maximum mix-up rate is best determined based on the purpose of the dataset. For instance, for clinical purposes, we can select high-quality participants for whom it is very unlikely that they reflect sample mix-ups using a maximum mix-up rate of 0.01% to 0.1%, which is the observed mix-up rate in diagnostic settings (Lippi *et al.*, 2017).

### 2.5 Calculating the predictive power of the sample mix-up method

To estimate the performance of *Idéfix*, we require a dataset in which it is known which samples are mix-ups and which samples are correct. This can be achieved by introducing fake mix-ups into a dataset. To get a reliable performance estimate, a considerable number of mix-ups will have to be introduced. However, since *Idéfix* expects that the majority of the sample mappings is correct, introducing a large proportion of sample mix-ups might underestimate the performance. Therefore, we created a separate training and testing dataset. We randomly sampled half of the Lifelines available samples (16 408) to use as training data. Thereafter, we induced 50% mix-ups in the testing dataset with the remaining samples (16 409). We used the model fitted on the training data to predict the mix-ups in the testing data. These predictions were used to establish an ROC curve and accompanying area under the curve (AUC). From the LLRs and ROC curve, we have extracted several thresholds. This was compared with the performance of a regular sex correspondence check in which the inferred sex from genotypes was compared with the reported sex to find mismatches. We also investigated the performance of a combined predictor that included both our PGS sample mix-up identification method and the sex correspondence check.

### 2.6 Simulating the relation between the number of used traits and the predictive power of PGSs

We have conducted several simulations to assess how the performance of *Idéfix* is affected by a varying number of traits and how the performance improves with an expected increase in predictive power of PGSs for the included traits (Supplementary Table S6). We therefore simulated PGSs and phenotype variables while explicitly maintaining the correlation structure of both the phenotypes, the covariates, the PGSs, and the correlation structure between these variables. Maintaining this correlation structure is important because in practice many complex traits are correlated, simulated un-correlated data would inflate the added value of additional traits. The data types of phenotypes (i.e. quantitative, binary, ordinal) are also maintained. To increase the number of traits, we duplicated the required part of the correlation structure while maintaining independence of traits between individual copies. To increase the explained variance of polygenic scores the squared correlations between all phenotypes and all polygenic scores were transformed by the inverse hyperbolic tangent function and multiplied by a predefined factor of 0.5, 1, 1.5 and 2. Thereafter, we transformed these values back using the hyperbolic tangent function to maintain the range from −1 to 1. For each dataset we performed five separate simulations in which we estimated the performance of *Idéfix* on 20 000 samples using the same approach that we used to estimate the performance of *Idéfix* in Lifelines. For all simulations, we calculated the predictive power using the method described in the previous paragraph and compared it with the performance between all simulated datasets.

*Idéfix* is implemented and run in the R programming language (version 3.6.1). Ordinal logistic regression models were fitted using the MASS package (version 7.3–51.6) (Venables and Ripley, 2002). ROCs and AUCs were calculated using the pROC package (version 1.16.2) (Robin *et al.*, 2011). For simulating datasets, we made use of the SimMultiCorrData package (version 0.2.2) (Fialkowski, 2018).

## 3 Results

### 3.1 Polygenic score-based sample mix-up identification

We have developed a sample mix-up identification method (*Idéfix*) that relies on the comparison of actual phenotypes to PGSs. Our method works by:

1. modeling the relationships between phenotypes and polygenic scores,
2. calculating the residuals of the provided samples and their permutations,
3. using a likelihood model fitted on the residuals for provided and permuted samples,
4. combining likelihood ratios over multiple traits,
5. estimating mix-up rates to select a subset of samples that adhere to a specified maximum mix-up rate.

We implemented our method in the R programming language (version 3.6.1). The installation and usage instructions for *Idéfix* is described on the wiki. (https://github.com/molgenis/systemsgenetics/wiki/Idefix).

### 3.2 Polygenic scores

We calculated PGSs for the 25 selected traits in the Lifelines dataset. To assess the predictive power of the PGSs for continuous traits $R^{2}$-values were calculated. For ordinal or binary phenotypes, we calculated spearman correlation and AUCs, respectively. These values indicate that for most traits, the performance is consistent with prior reported performance in literature. The explained variance ranges from 3.0% for the concentration of basophilic granulocytes to 33.9% for height. However, we observed that PGSs for body mass index (BMI) and educational attainment do not explain as much variance compared with previously reported values (11.9% and 5.3%, respectively). The explained variances for continuous traits are presented in Supplementary Figure S2. This figure also illustrates that, as expected, using a model that corrects for both sex and age increases the phenotypic variance that PGSs explain.

### 3.3 Predictive power of our method

We assessed the ability of *Idéfix* to discriminate between sample mix-ups and correct samples by executing the method we developed in the Lifelines dataset using the PGSs we calculated and the processed phenotypes. We fitted the regression and likelihood models on half of the samples in which we did not introduce artificial mix-ups. Subsequently, we applied these models on half of the samples in which we introduced 50% sample mix-ups. The measurements for the performance are therefore also suitable for situations in which a regular, moderate, percentage of mix-ups are expected. This approach allowed us to get an accurate measurement of the performance of our method for individual traits, as well as for the method overall. First of all, we see that AUCs for individual traits ranges from 0.50 to 0.66. In Supplementary Figure S3 is presented that these AUCs are positively correlated with the variance explained by PGSs, and that the predictive power of binary traits is, in general, less informative than that of continuous traits. The number of traits available for the samples in our dataset is presented in Supplementary Table S7.

**Fig. 2.**
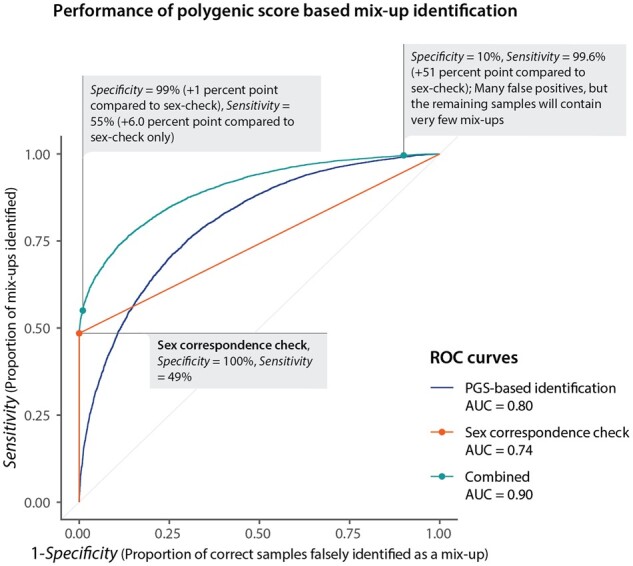
Performance of polygenic score-based mix-up identification. Performance of the polygenic score-based mix-up predictor (blue), the sex concordance check (sex correspondence check, orange) and a combined predictor (green) as illustrated by receiver operating characteristics (ROC). The *x*-axis indicates the proportion of correct samples that are falsely identified as a mix-up, named the false discovery rate (FDR), which corresponds to 1-specificity. The *y*-axis represents the proportion of mix-ups that are identified as mix-ups, named the true positive rate (TPR) or the sensitivity. Coordinates of one of the curves correspond to the specificity and sensitivity for a particular threshold of the predictor. Due to male–female imbalance in the dataset, the proportion of mix-ups identified as shown for the sex correspondence check deviates from the expected value of 0.5. Because of this deviation, the AUC is 0.74 as opposed to the expected AUC of 0.75 given an equal number of males and females

In Figure  2 is shown that the AUC for the polygenic score-based sample mix-up identification for 25 traits combined is 0.80. Using the AUC as a measure for performance, we also notice that our method performs better than the sex correspondence check which is able to identify 50% of samples without false positives if an equal number of males and females are present in the dataset. When combining the sex-check with our PGS-based sample mix-up identification the AUC increases to 0.90. It is conceivable that different thresholds are advantageous dependent on the intended application of the genetic data. For instance, GWASs profit from increased sample sizes (Canela-Xandri *et al.*, 2018), making lenient thresholds more suitable for this application compared with more stringent thresholds. Alternatively, when repurposing genetic data for clinical use more stringent thresholds are desired. In Figure  2, we estimate that the proportion of sample mix-ups is reduced 250-fold when selecting the 10% of participants for whom predicted phenotypes adhere best to the measured phenotypes.

### 3.4 Application in lifelines

Applying *Idéfix* to our entire cohort enabled us to identify a number of potential sample mix-ups. We note that there are four non-European samples that have considerably higher predictions to be mix-ups relative to other samples despite the fact that 98.2% of the analyzed samples are of Dutch ancestry. To ascertain that these findings are not ethnicity-driven, samples of European and non-European ethnicity were compared. This indicated that for non-European samples, there is on average not more evidence for classifying these as sample mix-ups (Welch's *t*-test, *P *=* *0.92), strengthening the support for the identified samples being mix-ups (Supplementary Fig. S4). By plotting the discordance between actual and predicted phenotypes, we can visually inspect how *Idéfix* identifies potential mix-ups (Supplementary Fig. S5). We can see that every sample of our top four predicted mix-ups shows systematic deviations for multiple traits, which indicates that not a single trait is driving the predictions. We also observe large deviations for height (*P*-values between 3.07 × 10^−3^ and 1.13 × 10^−8^). Given these observations, we conclude these are likely mix-ups.

Based on the number of mix-ups detected with a sex-check, it is expected that Lifelines still contains 56 sample mix-ups, constituting 0.15% of the total samples. In contrast, a diagnostic setting only allows for 0.01% to 0.1% sample mix-ups (Lippi *et al.*, 2017). We could use *Idéfix* to limit the mix-up rate in Lifelines to a mix-up rate of 0.01%. This allowed us to select 11 266 of 32 786 samples (34.4%) that are most likely correctly mapped. Herein, the expected number of mix-ups would be equal to the most stringent requirement for diagnostic use.

### 3.5 *Idéfix’*s performance increases when using more traits and with improved PGSs

**Fig. 3.**
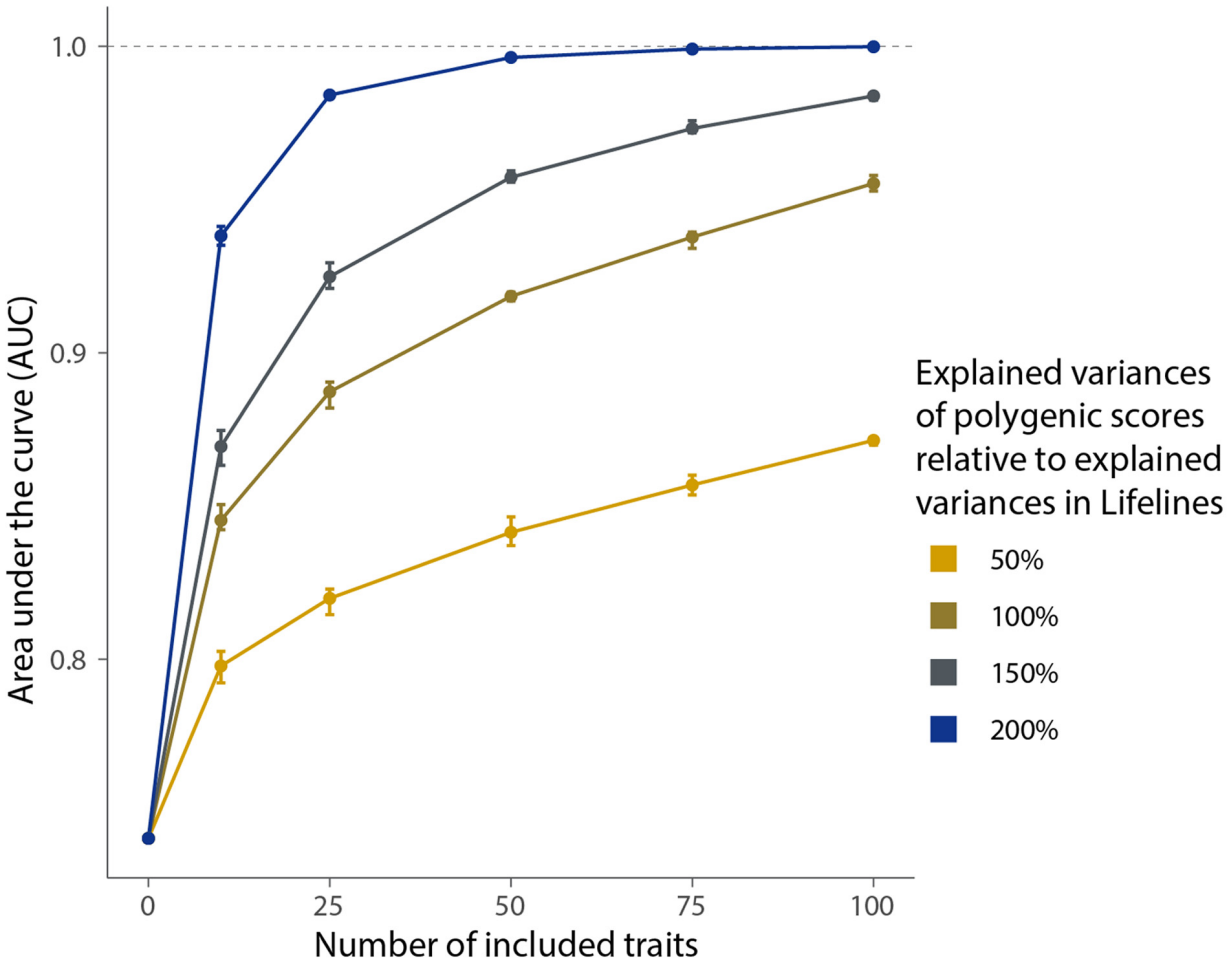
The increase in performance of *Idéfix* with an increase in the number of traits and an increase in power of PGSs. The figure shows that an AUC for 25 traits including the sex-check ranges from 0.82 to 0.98 when the explained variance of PGSs is varied from 50% up to 200%, relative to the actual explained variance of the PGSs in Lifelines. The points represent the mean for each simulated dataset over five iterations. Error bars represent the total range for the five iterations

Simulated datasets allow the performance of *Idéfix* to be assessed in diverse conditions: emulating larger biobanks with more traits or mimicking more powerful PGSs. In Figure  3 is shown that the AUC increases with an increase in number of traits and with an increased explained variance of PGSs. Simulations show that the performance of *Idéfix* reaches an AUC of 0.96 with 100 traits compared to 0.89 with 25 traits. We additionally show that after an increase of explained variance of all PGSs to 200%, the performance of *Idéfix* reaches an AUC of 0.98 and 1.00 for 25 and 50 traits, respectively.

## 4 Discussion

Sample mix-ups present a challenge for the repurposing of genetic data available in biobanks, as well as hindering the capability of association analyses to detect small genetic effects. Existing methods to resolve mix-ups are either not able to identify sample mix-ups within the same sex or require additional data like gene expression or methylation measurements. For most biobanks, such genomics data is usually not available. This was the motivating reason to develop *Idéfix*, a method that is able to identify sample mix-ups in biobanks while solely relying upon genetic data and a limited number of measured phenotypes, such as height, BMI, cholesterol and hair color.

We have shown that our method is capable of identifying sample mix-ups with high accuracy using both real data and simulated datasets. By accurately simulating datasets based on the observed correlations and distributions in our real dataset, we were able to model how increasingly powerful PGSs and additional traits affect the performance of *Idéfix*. With the increasing samples sizes of biobanks with genetic data, we can reasonably assume that the accuracy of PGSs in both European and non-European samples will improve in the near future and that they will be available for more different phenotypes. Based on this, we can conclude that the ability of *Idéfix* to identify sample mix-ups will improve considerably. This is extremely valuable for biobanks that wish to return information to their biobank participants, such as a pharmacogenetic passport or polygenic risk scores for certain diseases that can be (partly) prevented or intervened on by lifestyle modification. To do so, it is crucial to know that information that is being returned to individuals is accurate and our method *Idéfix* can help to ensure this to be the case.

Previously, it has been reported that predicting phenotypes based on genetic information with the aim of sample identification has remained a challenge (Cai *et al.*, 2017; Lippert *et al.*, 2017). However, the performance of the latter method was not clear (Erlich, 2017) and our approach is to select a high-quality dataset rather than sample identification. Here, we have leveraged a set of well-powered GWASs, in conjunction with a recent method for calculating PGSs, and a likelihood ratio framework to accurately weigh these PGSs to determine to what extent sample mix-ups can be identified. Most importantly, we developed this method because we aimed to identify a set of samples that very likely do not contain mix-ups and whom for instance can safely be returned pharmacogenetic results. Having such a sub-cohort of biobank participants available can, for instance, be very valuable for investigating the implications of pharmacogenetics among biobank participants alongside clinical implementation.

We do observe several avenues to improve our method. We have shown using both simulations and real data that our method performs best with well-powered PGSs (Supplementary Fig. S3). However, PGSs do not capture the full heritability of traits.

Furthermore, GWASs that identify variants associated with a particular trait which are the basis of PGSs are focused on common variants, while rare variants tend to have larger effect sizes (Marouli *et al.*, 2017). This is especially true for people with a rare monogenic disorder that effects multiple phenotypes. For instance, people with Marfan syndrome often present with medical complaints, but can live a relatively normal life (Marfan Syndrome, 2021). Since the incidence is between 1 in 5000 to 10 000, people with Marfan Syndrome are likely to be part of population-based biobanks. However, because they are typically tall and slender and often present bone, cardiovascular and ocular abnormalities they could be flagged as mix-ups since rare variants are not part of the PGSs that we used. While an increase in sample sizes will probably result in rarer alleles being discovered, the accuracy of PGSs can also be improved by integrating large-effect expression variants found in other ways as well (Smail *et al.*, 2020). Larger sample sizes also increase the power of GWASs to identify variants regardless of their effect size (Canela-Xandri *et al.*, 2018) and subsequently increasing the predictive power of PGSs yet more.

Another limitation of PGSs is that these are much less predictive for non-European cohorts (Duncan *et al.*, 2019). We show in simulations that a reduction of explained variance of 50% equates to a corresponding decrease in the performance of *Idéfix* (Fig.  3). This supports the need for large-scale GWASs in diverse human populations. A consequence of the less performant PGSs in non-European samples is that there will be less power to detect mix-ups, causing results to be differently distributed for these samples compared with European samples (Supplementary Fig. S4). We do expect that this limitation will be resolved when GWASs are performed on mixed ancestry in cosmopolitan cohorts. Until then, one possible solution is to run *Idéfix* separately for each ancestral population separately or using the recently proposed PGS-CSx that can handle mix-ancestry data (Ruan *et al.*, 2021).

In the past years, numerous biobanks have been established, each of these providing valuable resources on genotype data and a large number of phenotypes. This is a good starting point for our method. However, since the discovery and validation samples should be independent when calculating PGSs (Wray *et al.*, 2013), this poses a problem when biobanks are included in GWASs that are used for calculating PGSs, since these will then be biased for the included biobanks. However, we expect that the effect on the performance of *Idéfix* is limited. Since current GWASs are performed on many different cohorts, the effect of each individual cohort on the PGSs is limited.

Biobanks and studies often differ in the phenotypes that have been measured and that are available. With different independently inherited phenotypes being available for other biobanks, there is the opportunity for additional informative phenotypes to be included in *Idéfix*. However, this variability also limits the applicability to cohorts that have fewer phenotypes available since a limited number of phenotypes will limit the performance of our method, although it will still add to the performance of a sex-based concordance check. Moreover, due to the discrete nature of binary traits, these traits are, in general, less informative than continuous traits (Supplementary Note). Using highly heritable continuous traits is thus beneficial, although binary traits can also be a valuable aid while mix-up detection based on PGSs is not yet perfect. In addition, the first step of the method we developed is dependent in modelling the relationship between the measured phenotypes of individuals and the PGSs. Identifying such a relationship can be hindered by a lack of samples or can be biased when there are too many sample mix-ups present in the dataset.

Our method can be beneficial in a variety of scenarios. For instance, *Idéfix* is able to identify mix-ups irrespective of the proportion of males and females included in the study, whereas a common sex concordance check alone is only able to perform optimally with an equal number of individuals for both sexes. Our method relies on the availability of multiple traits in the biobank for which PGSs can be calculated. Furthermore, it is possible for our method to be expanded with additional traits when these become available in the future and it is expected that performance will increase dependent on the heritability of the trait. In the Lifelines cohort, 285 sample mix-ups have already been identified, 42% of which have been identified by utilizing pedigree information and ascertaining whether the genotype data is concordant with these reported familial relationships. These samples could not have been identified using sex-check alone, exemplifying the necessity of additional mix-up identification in biobanks wherein familial relationships cannot be used. Despite this thorough quality control in the Lifelines cohort, *Idéfix* has enabled us to identify 4 additional sample mix-ups with high confidence. Furthermore, it has allowed us to define a set of 34.4% samples wherein the estimated number of mix-ups is reduced 50-fold.

Currently, *Idéfix* is not yet able to identify every single sample mix-up: it can happen that for a certain sample the calculated PGSs for each of the 25 phenotypes are all quite average and that the observed, but incorrect phenotypic measurements behave similarly. In that case this sample is not yet flagged as a sample mix-up. However, using larger GWAS studies that lead to more accurate PGSs and by including more traits, we expect our method to help resolve most sample mix-ups in biobanks within the next few years.

## Supplementary Material

btab783_supplementary_data
