# Supplementary material for: Idéfix: identifying accidental sample mix-ups in biobanks using polygenic scores
Source: Bioinformatics. 2021 Nov 18;38(4):1059–66. doi: 10.1093/bioinformatics/btab783 (PMC8796367; doi:10.1093/bioinformatics/btab783)

Comparison of likelihood models

A: 1–15 / 25 traits

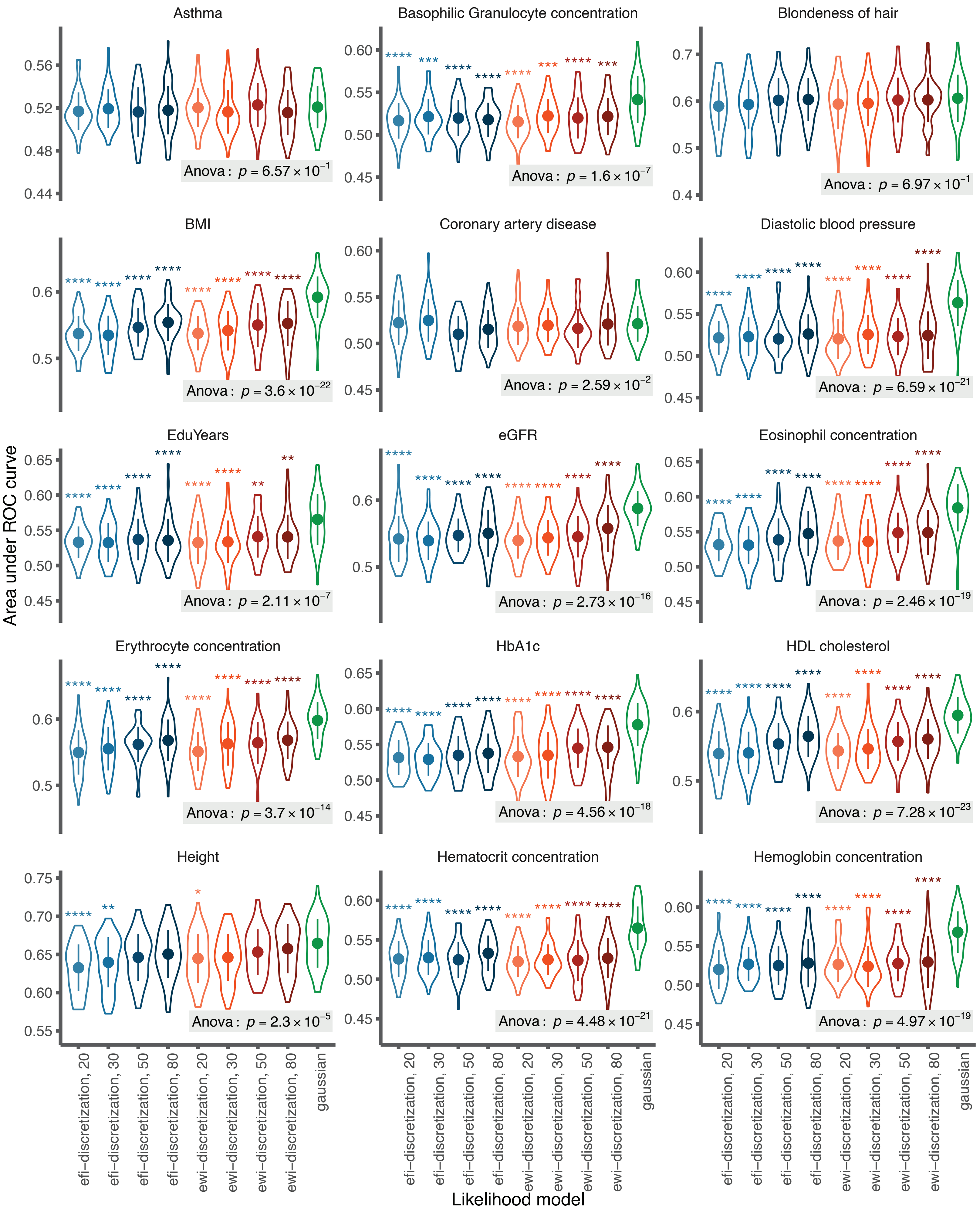

Comparison of likelihood models

B: 16–25 / 25 traits

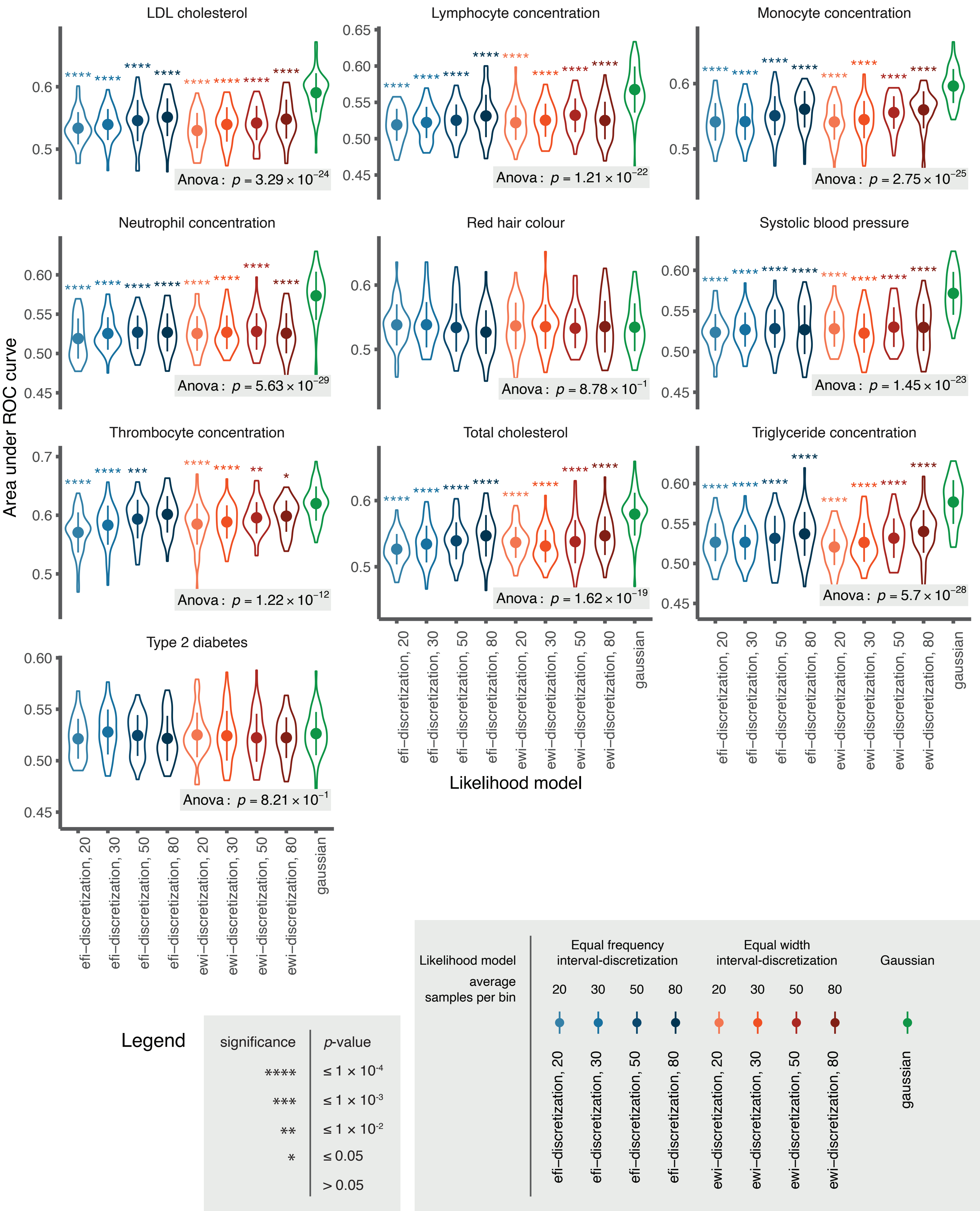

Supplement: btab783_supplementary_data [file btab783_supplementary_data.zip › Supplementary_Fig1_likelihood_model_comparison_20210104.pdf]
