# Supplementary material for: Idéfix: identifying accidental sample mix-ups in biobanks using polygenic scores
Source: Bioinformatics. 2021 Nov 18;38(4):1059–66. doi: 10.1093/bioinformatics/btab783 (PMC8796367; doi:10.1093/bioinformatics/btab783)
Supplement: btab783_supplementary_data [file btab783_supplementary_data.zip › Supplementary_Fig3_auc_per_trait_continuous_20210927.pdf]

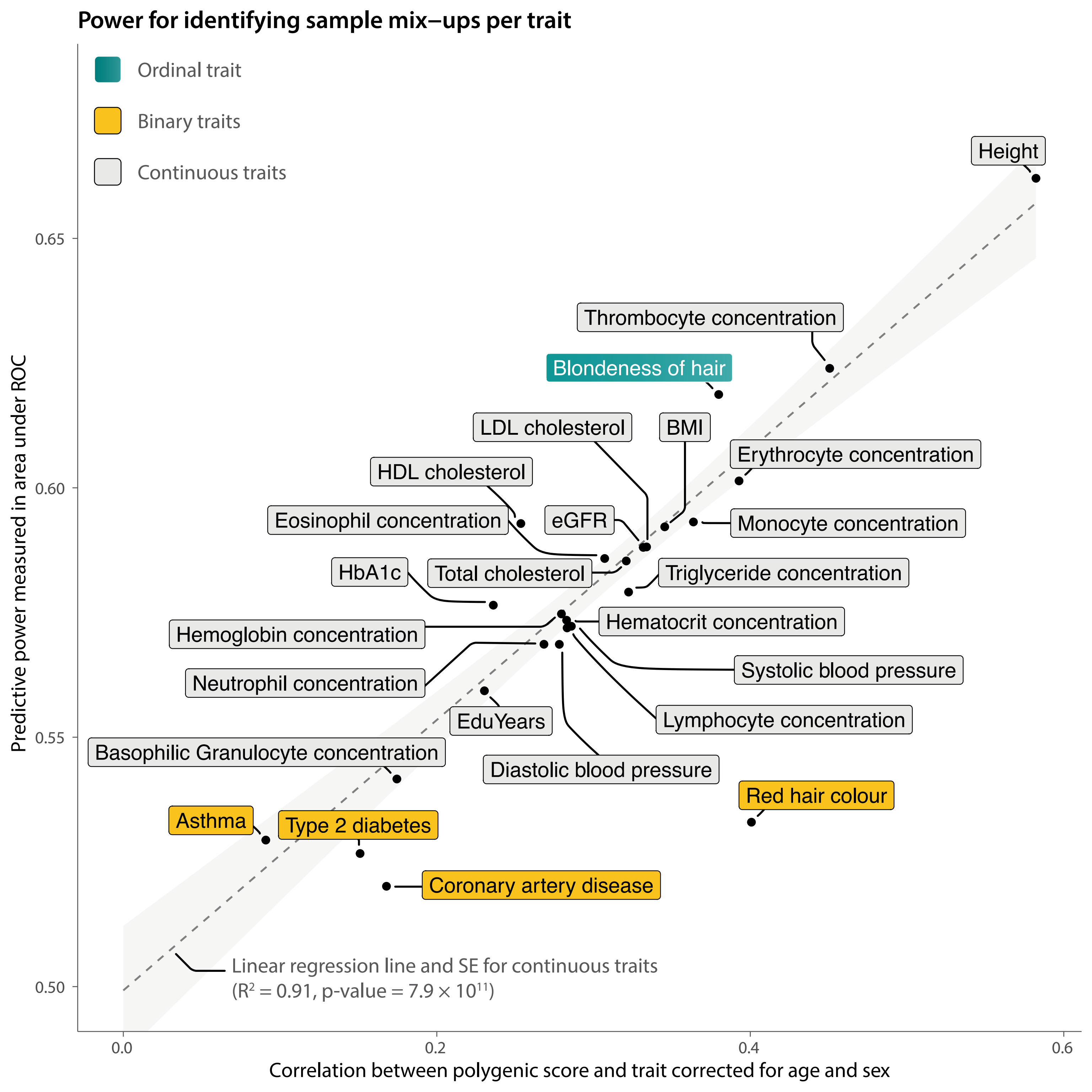

Power for identifying sample mix–ups per trait
Ordinal trait
Binary traits
Continuous traits
Height
Thrombocyte concentration
Blondeness of hair
LDL cholesterol
BMI
Erythrocyte concentration
HDL cholesterol
eGFR
Eosinophil concentration
Monocyte concentration
HbA1c
Total cholesterol
Triglyceride concentration
Hematocrit concentration
Hemoglobin concentration
Systolic blood pressure
Neutrophil concentration
EduYears
Lymphocyte concentration
Basophilic Granulocyte concentration
Diastolic blood pressure
Red hair colour
Asthma
Type 2 diabetes
Coronary artery disease
Linear regression line and SE for continuous traits
(R^2 = 0.91, p-value = 7.9 × 10^{11})
Predictive power measured in area under ROC
0.65
0.60
0.55
0.50
0.0
0.2
0.4
0.6
Correlation between polygenic score and trait corrected for age and sex
