# Supplementary material for: Idéfix: identifying accidental sample mix-ups in biobanks using polygenic scores
Source: Bioinformatics. 2021 Nov 18;38(4):1059–66. doi: 10.1093/bioinformatics/btab783 (PMC8796367; doi:10.1093/bioinformatics/btab783)
Supplement: btab783_supplementary_data [file btab783_supplementary_data.zip › Supplementary_Fig5_mixup-visuals_20210311-v2.pdf]

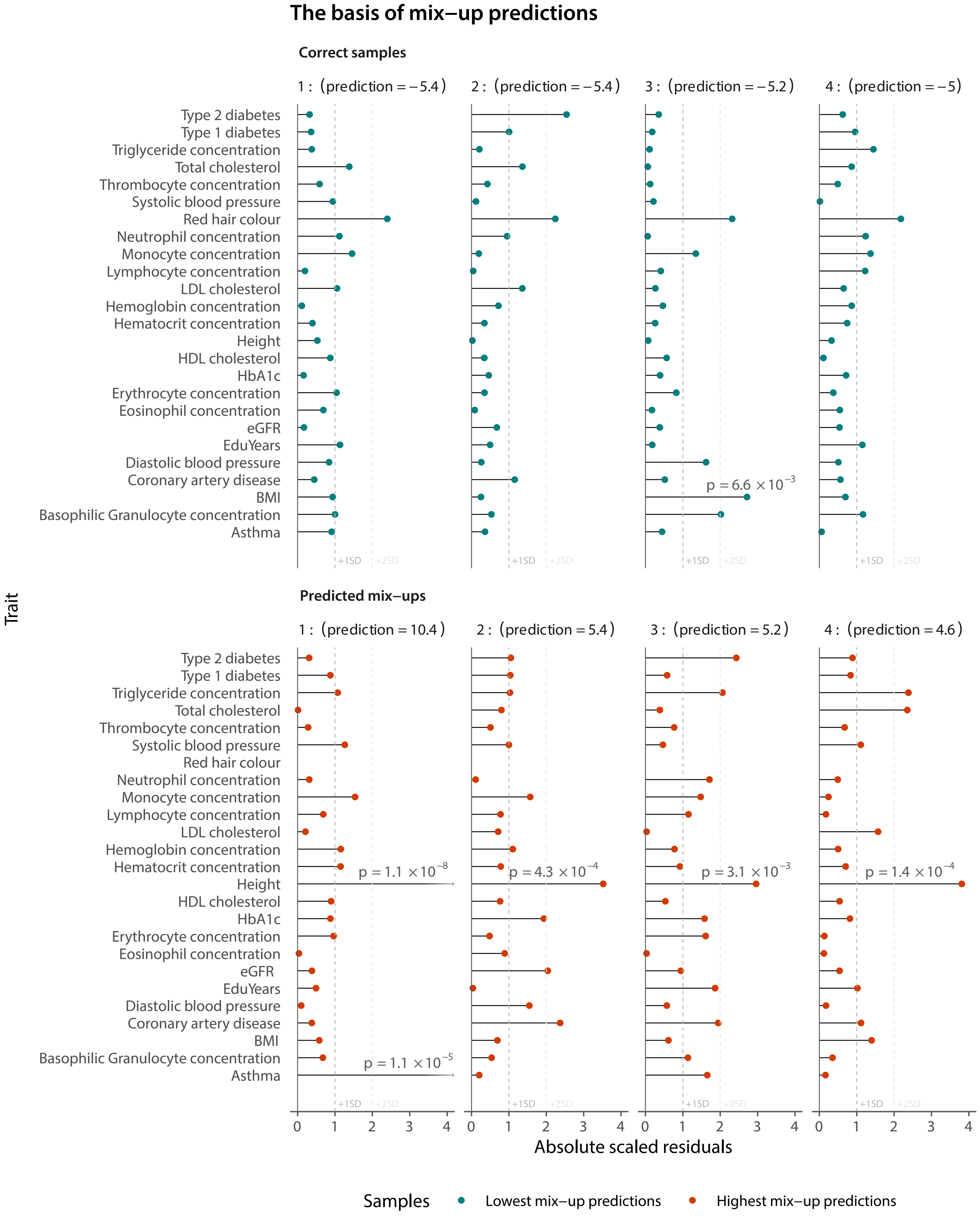

The basis of mix–up predictions
Correct samples
1 : (prediction = −5.4)
2 : (prediction = −5.4)
3 : (prediction = −5.2)
4 : (prediction = −5)
Predicted mix–ups
1 : (prediction = 10.4)
2 : (prediction = 5.4)
3 : (prediction = 5.2)
4 : (prediction = 4.6)
Type 2 diabetes
Type 1 diabetes
Triglyceride concentration
Total cholesterol
Thrombocyte concentration
Systolic blood pressure
Red hair colour
Neutrophil concentration
Monocyte concentration
Lymphocyte concentration
LDL cholesterol
Hemoglobin concentration
Hematocrit concentration
Height
HDL cholesterol
HbA1c
Erythrocyte concentration
Eosinophil concentration
eGFR
EduYears
Diastolic blood pressure
Coronary artery disease
BMI
Basophilic Granulocyte concentration
Asthma
p = 6.6 × 10−3
p = 1.1 × 10−8
p = 1.1 × 10−5
p = 4.3 × 10−4
p = 3.1 × 10−3
p = 1.4 × 10−4
+1SD
+2SD
Trait
0 1 2 3 4
Absolute scaled residuals
Samples
Lowest mix–up predictions
Highest mix–up predictions
