# Supplementary material for: Idéfix: identifying accidental sample mix-ups in biobanks using polygenic scores
Source: Bioinformatics. 2021 Nov 18;38(4):1059–66. doi: 10.1093/bioinformatics/btab783 (PMC8796367; doi:10.1093/bioinformatics/btab783)
Supplement: btab783_supplementary_data [file btab783_supplementary_data.zip › Supplementary_Note.pdf]

Supplementary note 1

Idéfix is implemented with different trait types in mind. Continuous traits, binary traits, and ordinal traits are supported. Here, we describe the added value of different traits towards predicting sample mix-ups. In supplementary figure 3 is shown how the variance of traits that is explained by polygenic scores attributes towards predictive power for identifying mix-ups. This is shown for 20 continuous traits, 4 binary traits and the single ordinal trait. The x-axis shows the correlation between polygenic scores and the actual phenotypes, represented by the square root of the variance explained, calculated according to *equation 1*.

$$\rho^2 = \frac{MSE_{partial} - MSE}{MSE_{partial}} \qquad 1$$

Herein ρ^2 represents the explained variance. MSE denotes the mean squared error of a model with the trait's actual phenotype as the dependent variable, and the polygenic score, sex and age as independent variables. $MSE_{partial}$ denotes the mean squared error of a partial model with only sex and age as independent variables. The y-axis represents the area under the receiver operating curve (AUC) for each of the traits for their ability to identify mix-ups. This is calculated by performing the Idéfix method per trait on all adult samples from Lifelines. The AUCs were calculated by comparing log likelihood ratios for the provided samples and permuted samples.

The figure shows that there is a linear relationship between square root of the explained variance and the AUC for the continuous traits (R^2 = 0.91, p-value = 7.9×10^{11}). The binary traits show to add much less information to the sample mix-up

prediction. This can be explained by the characteristics of the different trait types. Discrete variables can only depict a limited number of values. Just two in the case of the binary traits. Therefore, a subset of samples will by change share the same value. Samples in this set are thus not distinguishable based on this trait. The same is true for a sex correspondence check; given a set of males and an equal number of females, 50% of sample mix-ups by change occur between samples of the same sex. 50% of sample mix-ups will remain undetected. When the prevalence of one of two values is much larger than the other, sample mix-ups occur much more often between samples holding the same value, decreasing the mix-up detection even further. This has also depressed predictive performance for the binary traits. For instance, 13,187 samples have either brown, blonde or black hair colour, whereas only 380 samples (2.8%) have red hair colour. Thus, given *equation 2*, wherein $\mathbf{f}$ denotes the frequency of each of the values (0.028 and 0.972 for red and other hair colour respectively), the proportion of mix-ups that occur by chance between samples having different values, denoted by $swaps_{detected}$, is 0.054. Therefore, theoretically, a perfect prediction for red hair colour only allows for the detection of 5.4% of mix-ups, corresponding to an AUC of 52.7%.

$$swaps_{detected} = \sum_{i=0}^{n} (\mathbf{1} - \mathbf{f}_i) \times \mathbf{f}_i \qquad 2$$

True continuous traits theoretically depict an unlimited number of values. In practice, a limited number of decimal places are used for continuous variables. Therefore, by chance, samples can also share the same value for a continuous trait. Height for instance, is in our data commonly represented in centimetres, and occasionally in millimetres. Applying *equation 2* to the height trait, gives a value of 0.985. A perfect prediction would thus allow for the detection of 98.5% of mix-ups using only this trait,

corresponding to an AUC of 99.3%. Thus, in summary, continuous traits are more informative for mix-up prediction than binary traits, and, ideally, highly heritable continuous traits are used for the most effective mix-up detection. However, since *Idéfix* is not able to detect every single mix-up, binary traits can still be a valuable aid in detecting sample mix-ups. *Idéfix* weights every trait according to their predictive power, rendering uninformative traits harmless to the overall performance.
