# Supplementary figures and images for: Idéfix: identifying accidental sample mix-ups in biobanks using polygenic scores
Source: Bioinformatics. 2021 Nov 18;38(4):1059–66. doi: 10.1093/bioinformatics/btab783 (PMC8796367; doi:10.1093/bioinformatics/btab783)

Predictive power of adjusted continuous traits compared to literature

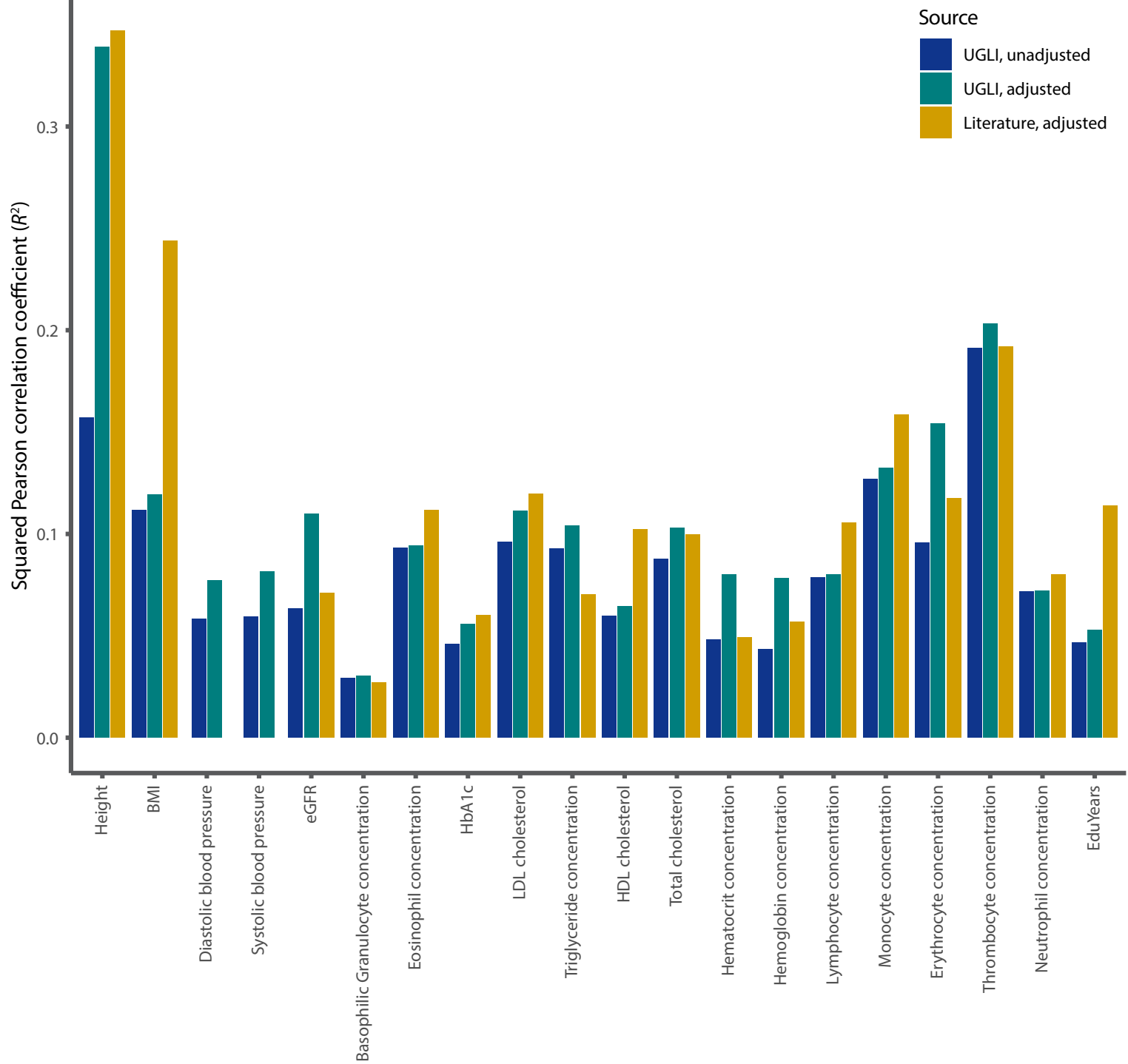

Supplement: btab783_supplementary_data [file btab783_supplementary_data.zip › Supplementary_Fig2_pgs_per_trait_continuous_20210127.pdf]

Comparison of European and non-European samples

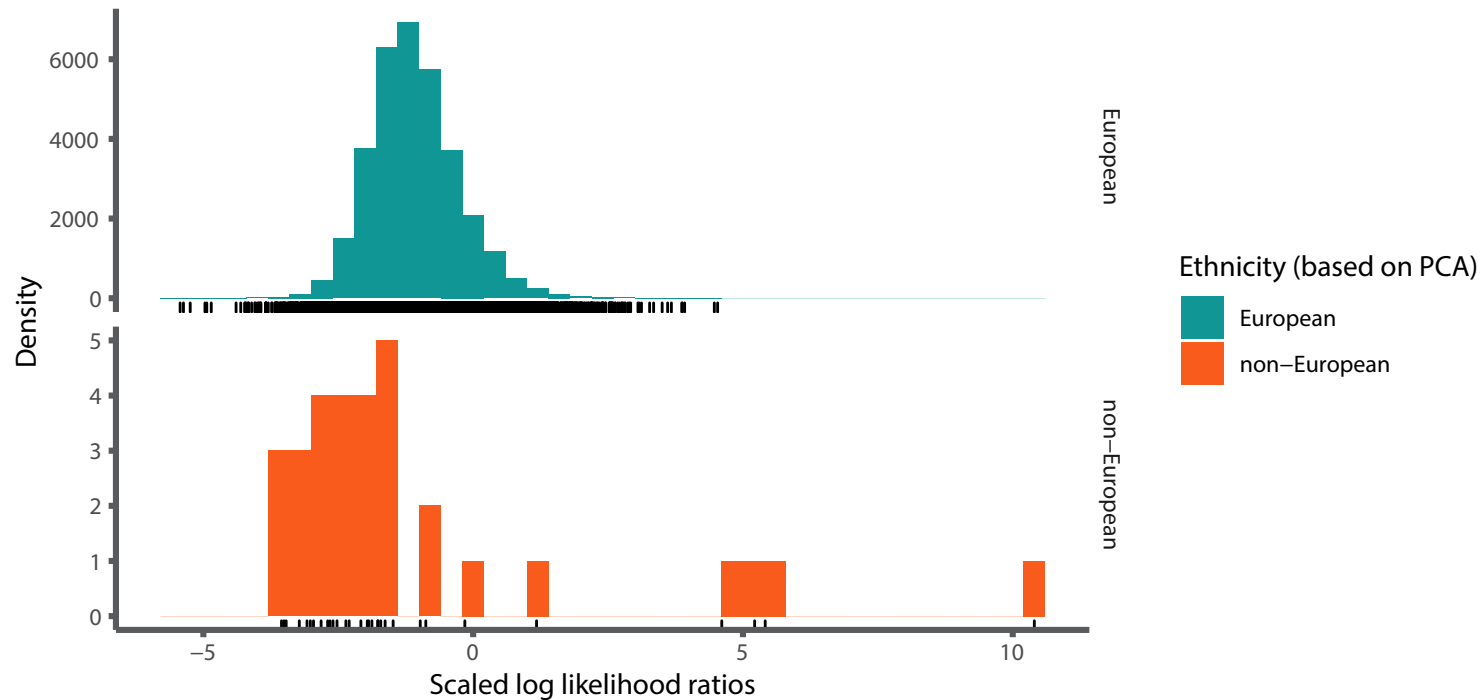

Supplement: btab783_supplementary_data [file btab783_supplementary_data.zip › Supplementary_Fig4_ethnicities_20210309.pdf]
